# Observational Study Regarding the Relationship between Nutritional Status, Dental Caries, Mutans Streptococci, and Lactobacillus Bacterial Colonies

**DOI:** 10.3390/ijerph18073551

**Published:** 2021-03-29

**Authors:** Eugen Silviu Bud, Cristina Ioana Bica, Oana Elena Stoica, Alexandru Vlasa, Daniela Eșian, Sorana-Maria Bucur, Anamaria Bud, Manuela Chibelean, Mariana Păcurar

**Affiliations:** 1 Faculty of Dental Medicine, University of Medicine and Pharmacy, Science and Technology George Emil Palade, 540139 Târgu-Mureș, Romania; Eugen.bud@umfst.ro (E.S.B.); cristina.bica@umfst.ro (C.I.B.); oana.stoica@umfst.ro (O.E.S.); Alexandru.vlasa@umfst.ro (A.V.); daniela.esian@umfst.ro (D.E.); manuela.chibelean@umfst.ro (M.C.); mariana.pacurar@umfst.ro (M.P.); 2 Faculty of Medicine, University Dimitrie Cantemir, 540545 Târgu-Mureș, Romania

**Keywords:** BMI, dental caries, children, obesity, DMFT, dmft

## Abstract

The prevalence of dental caries and obesity is high as both raise significant health problems. The objective of this study was to evaluate the relationship between dental caries, the number of salivary colonies forming units of Mutans Streptococci (MS) and Lactobacillus (LB), and the nutritional status in a group of children from Transylvania. This observational study used a sample of 154 school children, aged 9 to 12 years. The prevalence of caries was measured using the decayed, missing, and filled teeth index for deciduous teeth (dmft index) and for permanent teeth (DMFT index). Height and weight were assessed for each subject, and their body mass index (BMI) percentile was calculated. Salivary levels of Mutans Streptococci (MS) and Lactobacillus (LB) were determined using the CRT Bacteria Test from Ivoclar Vivadent. In our study, we found a positive association between the BMI percentile, MS count, LB count, tooth brushing frequency, and the incidence of dental caries in children aged 9 to 12 years old. Future preventive programs should include nutrition control in order to prevent both the apparition of dental caries and obesity in children.

## 1. Introduction

Despite many preventive measures, dental caries is one of the most common public health problems among children causing a social, physical, mental, and financial burden. Dental caries is the main cause of dental pain. It can determine mastication difficulties or it can have a profound impact on a child’s oral, general health, and quality of life [1].

The growth and development of a child are highly influenced by nutrition, thereby food consumption and eating behaviour throughout life has a great impact on general health, and on the health of the oral cavity. Diet has a strong impact on tooth mineralization and tooth development which could be affected by a nutritional imbalance [1].

Urbanization and economic development have led to changes in diet and lifestyle [2]. Consequently, obesity is the result of the chronic imbalance between energy intake and consumption. Studies have shown that most children become obese due to the consumption of foods with high caloric density, as well as a diet consisting of unhealthy fats or rich in carbohydrates [3].

Both obesity and dental caries are considered to be chronic, highly prevalent conditions, with potentially lifelong impacts on the lives of children and young people. They are multifactorial diseases with a complex etiology, both of which are associated with eating habits. There has been a growing interest in the relationship between dental caries and childhood obesity [4]. Although the dietary pattern of overweight and obese children may pose a risk for tooth decay, previous studies have demonstrated inconsistent and conflicting results [5]. Consequently, a diet rich in sugars and carbohydrates can be associated with various health problems such as tooth decay, obesity, and a poor-quality diet.

The association between different biological indicators of oral diseases and nutritional status has also been investigated. Lower stimulated salivary secretion rates, timing of tooth eruption, and different oral microbial profiles were observed in individuals with obesity. Risk factors such as bacterial counts of Mutans Streptococci (MS) and Lactobacillus (LB) in saliva have been correlated with initiation and progression of dental caries. MS has an important role in the onset of caries and LB is involved in the progression of caries [6].

MS is known to be the predominant microorganism in the etiology of dental caries. A considerable amount of research has been made regarding the role of this bacteria in causing caries and in predicting a higher caries risk [7,8]. Counts of salivary MS and LB have been extensively used to predict and monitor caries risk [9]. 

The early presence of MS in the mouth is connected with a high number of caries later in life and a high incidence of caries [8]. Oral bacterial diseases are not only caused by the MS but also by different types of bacteria [10].

Dental caries is a sugar and biofilm-dependent disease, since sugar is the element starting the entire process and providing the substrate for acidogenic species to produce organic acids. MS is among the acidogenic and aciduric species that plays a role in the build-up of biofilms [11]. Several studies indicate that the level of MS is not necessarily high in caries-associated biofilms, especially the microflora associated with non-cavitated stages of lesion formation [12].

The frequent consumption of high-calorie foods with carious potential are one of the many factors associated with obesity and tooth decay [13]. Consequently, numerous research have been carried out in order to identify the link between oral health and nutritional status. The patients’ general health condition is related to having no problems or diseases on all the anatomical structures, even involving the oral cavity functions or aesthetics. Today, great attention is focused on the prevention and maintenance of a high standard of oral hygiene and control [14,15], this study aims to investigate the relationship between dental caries and overweight in a group of school children in Transylvania, also evaluating other risk factors involved in the etiology of dental caries, namely tooth brushing frequency, the salivary MS and LB levels, and dental crowding. The identification of the potential risk factors before the onset of caries, is very important for planning and prevention strategies and focusing on the population that is most vulnerable to dental caries.

## 2. Materials and Methods

This observational study was conducted on a sample of 154 school children, aged 9–12 years, from Targu-Mures, Transylvania, Romania.

The study methodology was approved by the relevant local school authority, written informed consent was obtained from each patient that participated in this study. Furthermore, the ethics committee of the Algocalm Private Medical Center (Targu-Mures, Romania) approved the study (895/2 March 2020). Parents or guardians of all participating children have given written informed consent prior to the examination. Exclusion criteria were as follows:

- Children wearing orthodontic appliances;
- children taking medication;
- children with systemic conditions (gastroesophageal reflux disease, seizure, cancer, celiac disease).

The data collected was recorded in an examination chart for each subject, which included the name of the patient, date of birth, date of examination, sex, weight, and height. To avoid subjective errors, all measurements were performed by the same examinator and by one observer.

The frequency of daily tooth brushing was also recorded in the patient chart.

The non-invasive examination of the children took place in the school’s medical office. The objective examination was performed by direct and indirect inspection, using a dental mirror and by probing the dental surface, according to WHO recommendations [16]. All the teeth were examined in a systematic order, using the FDI numbering system of the teeth, under an adequate light and without prior dental radiographs [17].

Dental caries was determined based on visual-tactile criteria. The DMFT index for permanent dentition and the dmft index for deciduous dentition were used to describe the dental caries status for each child. Teeth with carious lesions were recorded as decayed, and also teeth with a softened floor and undermined enamel. As for dental units with temporary restorations, they were considered dental fillings. White spots were not noted as carious lesions and no X-rays were used in the study.

**Figure 1:**
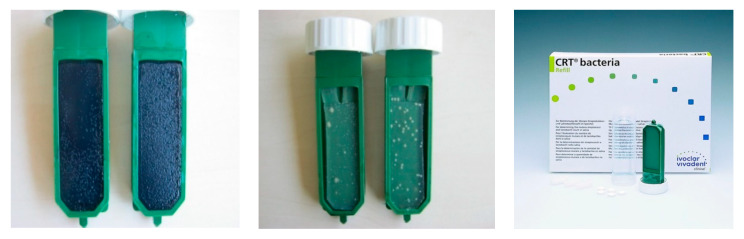
Colonies forming units (CFU) of Mutans Streptococci (MS) (**left**) and Lactobacillus (LB) (**middle**) and Caries Risk Test CRT (**right**).

The salivary bacterial count was determined using the CRT bacteria test (Ivoclar-Vivadent, Schaan, Liechtenstein) (Figure 1). The patients were asked to chew on a paraffin pellet that was included in the kit for 1 min and then they were instructed not to swallow and collect the saliva in a sterile container. Prior to the saliva collection patients were not allowed to eat, drink or brush teeth for at least 1 h, as recommended by the manufacturer. The saliva was inoculated on one agar plate for MS and another agar plate for LB, which were incubated at 37 °C for 48 h in the CRT incubator after adding a tablet of NaHCO3 to stimulate bacterial growth. We used a model chart provided by the manufacturer to assess the number of colonies forming units (CFU). The caries risk was scored as low, CFU less than 10^5^/mL and high, CFU greater than 10^5^/mL, as suggested by other authors [6,18,19,20].

The most common anthropometric index used to identify childhood obesity is the BMI or BMI for age. The percentile indicates the relative position of the child’s BMI among children of the same sex and age. Body weight and height were measured in light clothing and without shoes and BMI was then calculated using the following formula: Weight (kg)/height (m)^2^. The BMI for age percentiles (Table 1) was used and the sample was divided according to the international classification adopted by the Centers for Disease Control and Prevention [21].

For testing the normality of data we used the Kolmogorov Smirnov test, and applied statistical tests for independent parametric and nonparametric data. For the categorical data we used the chi square test, for the continuous data we used the Kruskal-Wallis test (comparison of groups). To identify the relational degree between certain parameters we used the Spearman and Pearson correlation. The chosen significance threshold was alpha = 0.05, and *p* was considered significant when *p* < 0.05.

## 3. Results

In our study group, 10 patients were underweight, 117 had normal weight, 27 patients were overweight, and no obese patients were found (Table 2).

There was no statistical significance regarding the patient’s sex and BMI percentile (*p =* 0.29).

There was a highly significant association (*p* < 0.0001) between the BMI percentile and DMFT, patients with a high BMI percentile, corresponding to overweight, have a significantly higher DMFT number than the other groups of patients.

**Figure 2:**
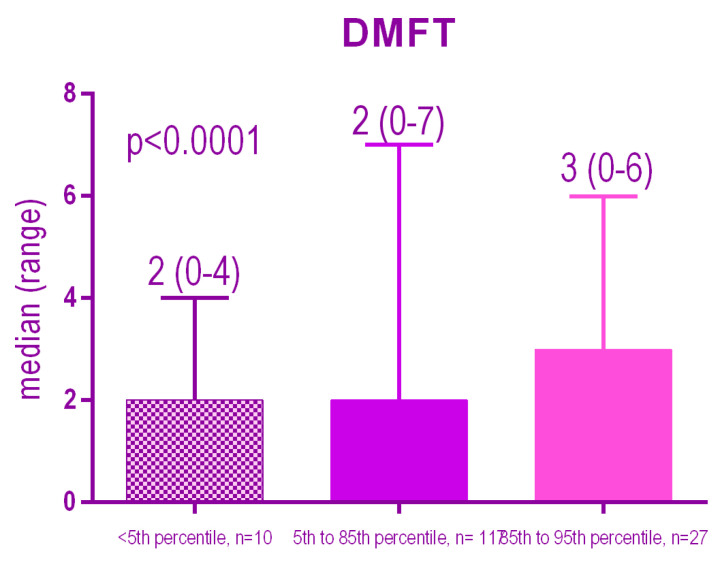
Distribution of the DMFT index according to the BMI percentile.

The Kruskal-Wallis test also indicates a highly significant difference (*p* < 0.0001) between the medians of the three groups, highlighting the fact that overweight patients (group 3) have a significantly higher median DMFT than normal or underweight patients (Figure 2).

There is a highly significant association (*p* < 0.0001) between the BMI percentile and the dmft number. Patients with a high BMI percentile have a significantly higher dmft compared to other groups of patients.

**Figure 3:**
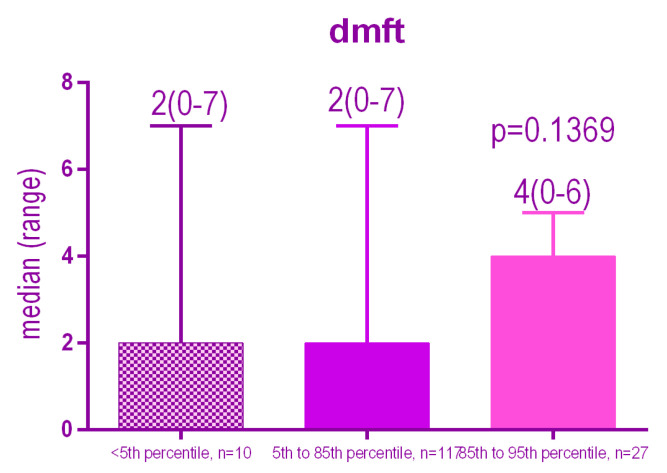
Distribution of the dmft index according to the BMI.

The Kruskal-Wallis test indicated that there is no significant difference (*p* = 0.1369) between the medians of the three groups (Figure 3).

**Figure 4:**
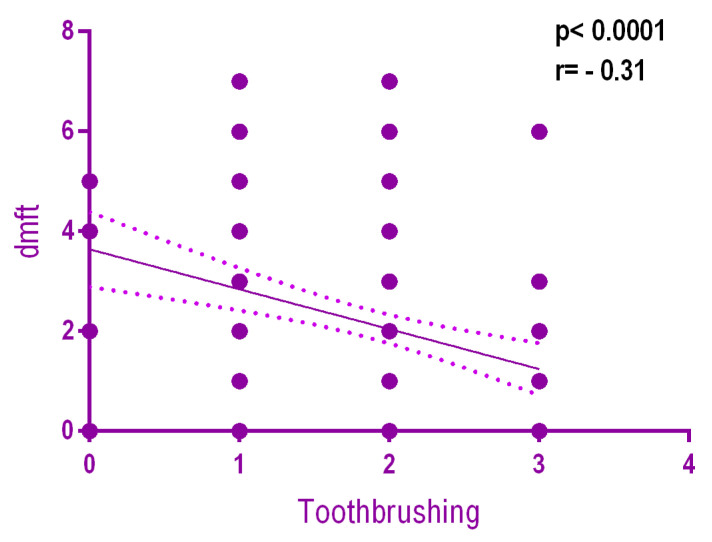
Relationship between dmft and toothbrushing frequency.

There was a weak but statistically significant negative relationship (*r* = −0.31, *p* < 0.0001) between dmft and the frequency of toothbrushing. The more frequent the toothbrushing, the lower the dmft (Figure 4).

**Figure 5:**
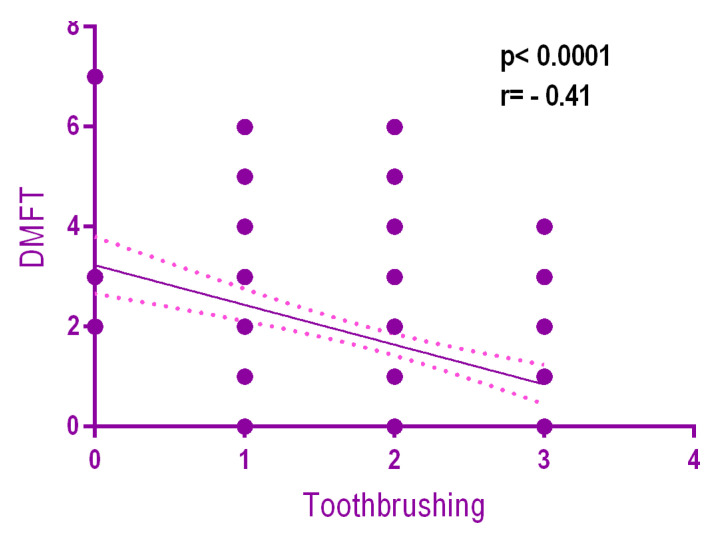
Relationship between DMFT and toothbrushing frequency.

There is a weak but statistically significant negative relationship (*r* = −0.41, *p* < 0.0001) between DMFT and the frequency of toothbrushing. The more frequent the toothbrushing, the lower the DMFT (Figure 5).

**Figure 6:**
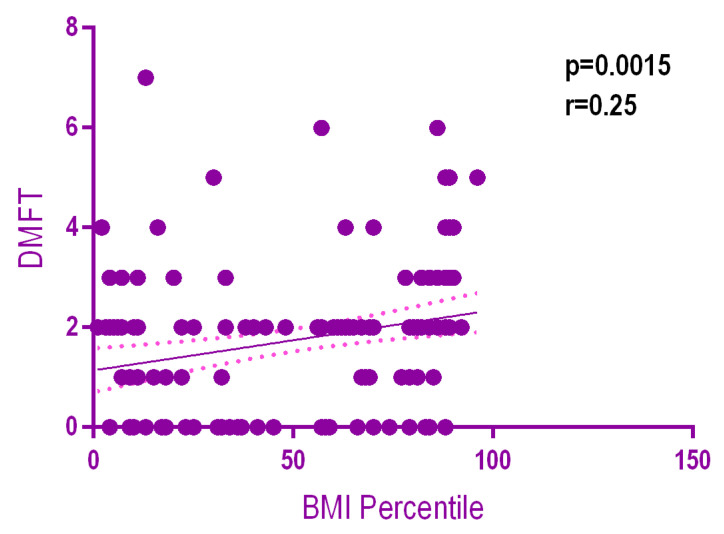
Relationship between the DMFT and BMI percentile.

**Figure 7:**
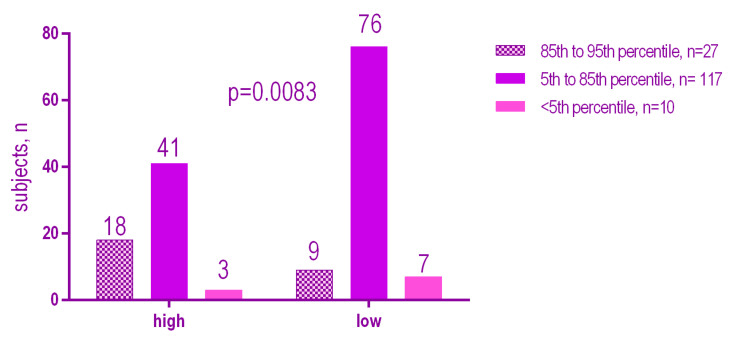
Mutans Streptococci levels according to the BMI.

**Figure 8:**
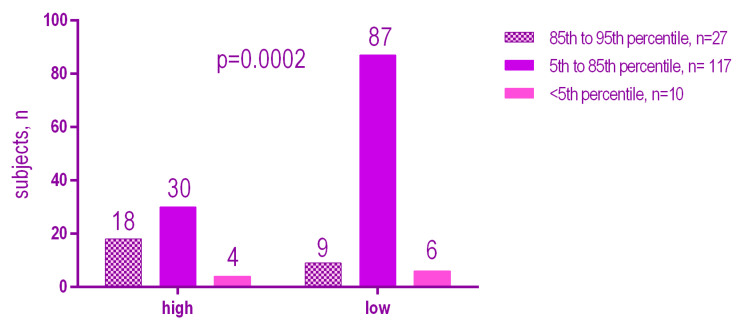
Lactobacilli levels according to the BMI.

There was a weak but statistically significant positive relationship (*r* = 0.25, *p* = 0.0015) between the DMFT and BMI percentile. The higher the BMI value, the higher the DMFT (Figure 6). There was no statistically significant positive relationship (*p* = 0.0083) between the Mutans Streptococci levels according to the BMI (Figure 7). On the other hand there was a significant positive relationship (*p* = 0.002) between Lactobacilli levels according to the BMI (Figure 8).

The results showed a significant association between the high presence of MS (high) (*p* = 0.0083), and LB (high) (*p* = 0.0002) and an increased BMI percentile. Overweight patients have a higher level of MS and LB than normal or underweight patients.

## 4. Discussion

Due to the multifactorial nature of dental caries, the salivary properties, oral hygiene frequency, and nutritional status of the patients should be recorded in order to establish their roles as risk factors in the development of dental caries [6].

Several studies have shown that the prevalence of overweight and obesity among children from both developed and underdeveloped countries is increasing and this issue is becoming a public health concern [22]. It is plausible that a high BMI can be harmful to the oral health of a child [23].

Dental and oral health are an inseparable part of overall health, as they would affect the quality of life, appearance, communication, and proper nutrition. Thus, good practices of oral care and hygiene throughout life are the basis of continuous optimal health during childhood, adolescence, and adulthood [24].

Changes in the environment that promote a sedentary lifestyle and high consumption of foods and beverages with an increased content of carbohydrates, have led to an increase in the number of overweight and obese children. To establish that both dental caries and childhood obesity can be targeted by a common risk factor, it is necessary to determine whether the two diseases are really associated [1].

In our study, which used three risk factors to predict dental caries, we found a positive association between the BMI percentile and the incidence of dental caries in children aged 9–12 years. It is necessary to analyze multiple risk factors, such as oral hygiene, bacterial counts, and tooth brushing frequency.

Similar results to our study were found by Touran Shahraki et al., One thousand two hundred and thirteen (1213) children were included in this study, of these, 20.8% were underweight, 66.3% were normal weight, 7.8% were registered as overweight, and 5.1% were distributed to obese children. A significant association between BMI and DMFT (*p* = 0.005) was found [25]. Hong et al., found also in his study that childhood obesity was significantly associated with carious lesions in permanent dentition [26]. At the same time, Gerdin et al., Powell et al., and Yao et al., found that obesity or an unhealthy body mass index, was linked to an increased number of carious lesions [27,28,29]. This can be caused by an increased pro-inflammatory status maintained by high levels of cytokines associated with obesity [30].

Moreover, in a study conducted by Elangovan et al., on 510 children, it was found that the prevalence of carious lesions was higher in obese children than in other BMI groups. In this context, the tooth decay score was higher as the BMI for age increased, although this was not statistically significant [31].

In contrast to these findings, other research has reported little or no association between body mass index and the carious disease [27,32,33]. The study conducted by Sudhakar et al., proved to be a negative correlation (*r* = −0.14) between BMI and DMFT [34]. On the other hand, Scheller et al., found no association between BMI and dmft [35]. Benzian et al., and Bafti et al., also found an inverse relationship, reporting an association between underweight and a high score of the dmft index, although it should be noted that these studies were not conducted in Western countries [36,37]. Similarly, Sanchez-Perez et al., examined 110 Mexican children, ages 7–11. The researcher did not find a significant association between tooth decay in mixed dentition and BMI. Thus, the author found that the prevalence of dental caries was not significantly different among BMI categories. At the same time, this discrepancy may be due to the differences between the study models [38]. Our results showed a positive correlation between BMI and high levels of MS and LB. Similar results were found also by Araujo et al., in his research, adolescents with overweight or obesity showed the highest percentages of MS and *Bifidobacteria* than normal-weight ones. His results are indicating a possible interaction between oral bacteria communities and weight gain [39].

The habit of ingesting high levels of sugary foods and drinks may explain the high levels of MS in overweight children.

However, in another study, adolescents with excess weight but free of dental caries showed the highest percentage of MS in saliva compared to normal-weight ones [40].

In this context, previous studies showed that sucrose consumed several times a day in small quantities could induce changes in the salivary microbiome and MS levels [41]. Obese women (BMI > 30 kg/m^2^) had significantly higher numbers of MS than normal-weight women (*p* < 0.01). Furthermore, there was a small but significant correlation between MSi and BMI, but no correlation between LB and BMI [42].

A study from Sweden that investigates the association between salivary counts of MS and children’s weight status, found that medium-high counts of MS were positively associated with a higher BMI Z-score (OR = 1·6; 95% CI 1·1, 2·3). Positive associations were also found between medium-high counts of MS and more frequent meals per day (OR = 1·5; 95% CI 1·1, 2·2), greater percentage of sugar-rich foods consumed (OR = 1·1; 95% CI 1·0, 1·3), and female sex (OR = 2·4; 95% CI 1·1, 5·4) [16].

Similar results to the previous study were found in an indigenous child population from Australia, in which high salivary loads of MS and LB were significantly associated with dental caries experience, even after adjusting for other salivary characteristics (pH, flow, buffering capacity), tooth brushing frequency, and soft drink consumption. Such findings are well known from previous studies but have not been previously demonstrated in a remote, indigenous community [43].

A similar result was found in a study on children from private schools in Accra, Ghana that compared the prevalence of MS in obese and non-obese school children and assessed its association with dental caries. There was a significantly higher prevalence of MS among the obese children (41.3%; 95% CI, 30.6–52.7%) compared to the non-obese (26.0%; 95% CI, 7.6–37.5%), *p* = 0.033. Caries prevalence was similar in the obese 14.9% and non-obese 15.1%, but there was no significant association between MS infection and the dental caries [44].

In contrast to these results, another study conducted by Raju on 900 Finnish children, aged 11–14 years, found the decrease in the core bacteria (*Veillonella*, *Prevotella*, *Selenomonas*, and *Streptococcus*) in overweight and obese children [45].

In Finland, most of the children and adolescents have good oral health, and public oral healthcare services are freely available for individuals under 18 years of age. Individual preventive efforts are provided for certain age groups and declining trends in the caries occurrence rate has been seen. Moreover, in Finland, 11 and 12 year old children are unlikely to be dieting, smoking, and consuming alcohol, which are factors that could influence the results. Children in Finland receive healthy lunches and snacks provided by the school system as part of their free education [45].

Regarding the frequency of tooth brushing, the results obtained are consistent with many studies in the literature, indicating a statistically significant association between the frequency of brushing and the number of caries, both in the case of temporary and permanent teeth [46,47,48].

### 4.1. Practical Application

Being overweight can be considered a risk factor for tooth decay, so the dentist should provide information and advice on healthy nutrition by establishing and implementing effective prophylaxis programs.

Pediatric patients require a comprehensive multidisciplinary approach, both by pediatricians, endocrinologists, nutritionists, and the dentist to prevent obesity and carious lesions, both of which have a negative impact on the patient’s health.

### 4.2. Limitations of the Study

The results of this study can be seen with certain limitations, namely the rather small number of the study group and the specific population, school children in Targu-Mures. Although the specific population may make it difficult to generalize the results to other geographical regions, the present study contributes to the increase of reference data in the literature, supporting future research in this field. Given that no radiographs were used in this study, DMFT/dmft values could be slightly underestimated.

Future studies, with a longitudinal evaluation, on larger patient groups are needed, taking into account other variables with a potential role in caries etiology (social, environmental, economic, etc.) in order to decrease caries prevalence, according to WHO requirements for 2020.

The study included a limited number of children due to the current COVID-19 pandemic, which made it impossible to continue the research in schools.

## 5. Conclusions

In our study, overweight children have a significantly higher DMFT and dmft caries index compared to the underweight and normal weight children. The rate of dental caries in the permanent dentition of children aged 9–12 increases with BMI and the dmft index increases less.

The frequency of brushing is an associated factor in the etiology of caries, as patients with poor oral hygiene have higher values of the caries index (DMFT and dmft).

There was a strong correlation between the high MS and LB and high prevalence of dental caries. The presence of high bacteria counts can be confidently used to predict dental caries in young children in this population.

Dental caries and overweight have common risk factors that can persist into adulthood and increase the risk of chronic disease, so the pediatric dentist might be in an optimal situation to improve oral health and reduce the risk factors for overweight and obesity.

The relationship between dental health, BMI, and salivary bacteria was not previously studied on the Romanian population. The results of the present study are only indicative, but not confirmatory. The multifactorial etiology of both diseases requires further research, on larger samples, with a longitudinal approach, rather than a cross-sectional one.

## Figures and Tables

**Table 1:** Body mass index (BMI) percentile, indicator of the nutritional status of children aged 2 to 20 years (CDC, 2000) [18].

Nutritional Status	BMI Percentile Range
Obesity	BMI ≥ percentile 95
Overweight	85 ≤ BMI < percentile 95
Normal weigh	5 ≤ BMI< percentile 85
Underweight	BMI < percentile 5

Statistical analysis was performed with the GraphPad Prism software, v6.0 (GraphPad™, San Diego, CA, USA).

**Table 2:** Index for permanent teeth (DMFT) score, index for deciduous teeth (dmft) score in underweight, normal, and overweight patients.

		Group 1 <5th Percentile, *n* = 10	Group 2 5th to 85th Percentile, *n* = 117	Group 3 85th to 95th Percentile, *n* = 27	*p*-Value
Sex	M	2	53	11	0.29
	F	8	64	16	
DMFT	0	0	39	1	<0.0001
	1–3	7	73	15	
	4–6	2	5	11	
	>6	0	2	6	
	Total	21	165	86	<0.0001
Dmft	0	2	34	4	<0.0001
	1–3	6	66	5	
	4–6	1	15	18	
	>6	1	2	0	
	Total	24	214	31	0.1369

## Data Availability

Not applicable.
